# Meet the *PCP* Editor—Rajeev K. Varshney FRS

**DOI:** 10.1093/pcp/pcad064

**Published:** 2023-06-20

**Authors:** Rajeev K Varshney

**Affiliations:** WA State Agricultural Biotechnology Centre, Centre for Crop and Food Innovation, Murdoch University, Murdoch, WA 6150, Australia

**Figure:**
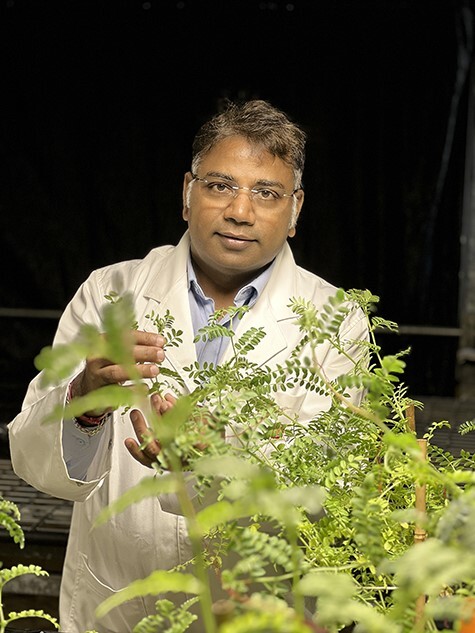
**Photo:** Rajeev Varshney, an Editor with *PCP* since 2022 and responsible for handling papers mostly on abiotic stress, crop traits, breeding and genomics.

Rajeev K. Varshney attended Aligarh Muslim University, India, where he completed his undergraduate studies (B.Sc. Honors in Botany) in 1993 and M.Sc. in Botany (specializing in Genetics, Plant Breeding and Molecular Biology) in 1995. He obtained his doctoral degree in Agricultural Botany (Molecular Biology), in 2001, under the guidance of Prof. P. K. Gupta and Prof. P. C. Sharma at Chaudhary Charan Singh University, India. From 2001 to 2005, Rajeev worked as a Postdoctoral Research Scientist on the structural and functional genomics of barley and on comparative genomics of cereal grasses, under the supervision of Prof. Andreas Graner at the Leibniz Institute of Plant Genetics and Crop Plant Research (IPK), Germany. Subsequently, Rajeev joined the International Crops Research Institute for the Semi-Arid Tropics (ICRISAT), India, as a Senior Scientist from September 2005 to February 2022, serving in various roles: Principal Scientist, Director-Center of Excellence in Genomics & Systems Biology, Research Program Director (RPD)-Grain Legumes, RPD-Genetic Gains and RPD-Accelerated Crop Improvement Program. While working at ICRISAT, he also held dual appointments with the CGIAR Generation Challenge Programme as a Subprogram Leader for Genomics towards Gene Discovery (during 2007–2013), hosted at the International Maize and Wheat Improvement Center (CIMMYT), Mexico. Additionally, he has held several adjunct or visiting professorships at the University of Western Australia, the Guangdong Academy of Agricultural Sciences and the Beijing Genomics Institute in Hong Kong, among others. In February 2022, Rajeev joined Murdoch University as Director of the Western Australian State Agricultural Biotechnology Centre and Director of the Centre for Crop and Food Innovation, where he also holds the position of International Chair in Agriculture and Food Security with the Food Futures Institute. Rajeev was elected a Fellow of the Royal Society (FRS) in 2023 and is also an elected fellow/Academician for about 10 other science and agriculture academies/societies in India, Germany, the USA and Africa. Rajeev is a highly prolific author and a Clarivate Highly Cited Researcher for nine consecutive years (since 2014), and his research work has been recognized with >30 prestigious awards worldwide, including the Shanti Swarup Bhatnagar Prize, the Rafi Ahmed Kidwai Award (Government of India), the International Crop Science Award (Crop Science Society of America) and the Qilu Friendship Award (China). Rajeev currently serves as a handling editor for *Plant & Cell Physiology (PCP)*, since 2022, and mostly handles papers in crop traits and genomics.

## Why Did You Decide to Study Plants?

Growing up in a small town in Uttar Pradesh state of India, I had aspirations of becoming a space scientist, inspired by my love of reading comics. However, being in a primarily agrarian economy in India, agriculture impacted the lives of everyone, including myself. This led me to develop an interest in plant sciences, specifically the science behind genes. Later, during my postdoctoral tenure at IPK, Germany, I attended the ‘From Green Revolution to Gene Revolution’ conference in Bologna, Italy, where I had an opportunity to meet renowned agricultural scientists such as Prof. Norman Borlaug, Prof. M. S. Swaminathan, Prof. Gurdev Khush and Prof. Mike Gale, among others. Prof. Borlaug in his speech challenged the next generation of scientists to embrace new tools and technologies to address food security issues in developing countries. This motivated me, as an early-career researcher, to focus on translational aspects of upstream research to develop better crop varieties with improved yield and nutrition. With this mission in mind, I started my research group, and Center of Excellence in Genomics (later rebranded as the Center of Excellence in Genomics & Systems Biology) at ICRISAT, India, with the aim of bringing prosperity to the lives of legume-growing smallholder farmers in Asia and Africa who had not yet benefited from the Green Revolution. I feel proud to say that together with colleagues and collaborators, we didn’t just generate genomic resources in the so-called orphan tropical crops but also integrated advanced genome discoveries to develop climate-change-ready, high-yielding and nutritious legume crop varieties. In addition to refining the breeding pipeline, we addressed last-mile delivery issues through the Tropical Legumes projects by establishing and strengthening seed systems in 13 African and two Asian countries. In brief, the outputs of this project touched about 25 million lives in Africa and Asia.

## What Are the Main Research Avenues Being Pursued in Your Laboratory?

The abundance of DNA sequence information has provided opportunities for crop improvement by identifying genes and molecular markers associated with diverse agronomic traits. To achieve this, over the last 18 years, my research group has collaborated with partners worldwide to create extensive genomic resources, such as draft genome assemblies, thorough genetic and physical maps, thousands of simple sequence repeat markers, millions of single nucleotide polymorphism markers, and various high-throughput and cost-effective marker genotyping platforms. Importantly, we have successfully sequenced the reference genome assemblies for >14 plant species, including pigeon pea (Varshney et al., 2012), chickpea (Varshney et al., 2013), groundnut (Bertioli et al. 2019, Zhuang et al. 2019), pearl millet (Varshney et al., 2017), sesame (Wang et al. 2014), mungbean (Kang et al. 2014), pea (Yang et al. 2022), adzuki bean (Yang et al. 2015), longan (Lin et al. 2017), Jatropha (Ha et al. 2019), soybean (Valliyodan et al. 2019) and celery (Song et al. 2021). We have also been involved in resequencing many of the crop germplasm accessions contained within the ICRISAT Genebank depository; for instance, as part of a collaborative effort, we generated genome sequence and phenotypic data for about 3,366 chickpea accessions (Varshney et al. 2021b). Based on these genomic resources, we identified genetic loci associated with 30–50 traits in chickpea, pigeon pea and groundnut. Subsequently, we worked with breeders in many developing countries to deploy fast-forward breeding approaches (Varshney et al. 2021a), resulting in the development of several improved lines and more than a dozen improved legume crop varieties that have been released in India and Ethiopia.

My research group at Murdoch University is currently working on improving wheat, legume and horticultural crops for a range of agronomic and abiotic stress tolerance traits by developing and deploying novel genomics and pre-breeding approaches such as pangenomics, haplotype cataloging, functional genomics and artificial intelligence (AI) approaches.

## What Is the Best Thing about Being an Editor with *PCP*?

In my opinion, it is evident that as researchers, we ought to publish our work in journals that are affiliated with an esteemed society whenever feasible, so that the funds we allocate toward publication aid in advancing plant science both domestically and globally. *PCP* has a distinguished track record of publishing top-notch papers in the plant sciences. As an example, some of the recent articles published in the journal highlight innovative research, such as the integration of genomic features using a supervised machine learning technique to enhance the accuracy of predicting transcription factor–binding sites in plants, as described by Rivière et al. (2022). Another article by Maeda et al. (2023) explores the potential of single-cell RNA sequencing in identifying various specialized cell types in *Arabidopsis* leaf tissues, including glucosinolate-producing and idioblast myrosin cells. Over the years, I have collaborated with various national and international organizations worldwide, and for me, serving as an editor with *PCP* presents an additional opportunity to remain connected and learn more from the plant cell and physiology research community.

## What Tips Would You Give Early-Career Plant Researchers?

Currently, there are nearly one billion people worldwide experiencing hunger, with projections indicating a sharp increase by 2050. This situation is further exacerbated by the annual loss of crop yields due to climate change. I believe that all disciplines of plant sciences are equally essential, as they help us understand biological processes and enable us to improve plant growth. For developing countries, agriculture is crucial since it remains the primary means of livelihood for the majority of the population. Aspiring researchers and early-career professionals should prioritize addressing critical challenges, such as how to feed the expected nine billion people by 2050. This pursuit presents an abundant opportunity to make a significant impact and is likely to be fulfilled in the years ahead. Moreover, young researchers must demonstrate commitment and work hard to attain their goals. While life may present obstacles and unexpected hurdles, it is vital to maintain focus and adjust the approach rather than the objective. I am enthusiastic about seeing more young researchers focusing on agriculture and life sciences, especially given their significance highlighted by the corona virus disease (COVID-19) pandemic, climate change and countries’ conflicts. The importance of agriculture is well articulated by Prof. M. S. Swaminathan, the Father of the Indian Green Revolution who said, ‘If Agriculture Goes Wrong, Nothing Will Go Right’.
